# Evaluation of Novel Stereotactic Cannula for Stem Cell Transplantation against Central Nervous System Disease

**DOI:** 10.1155/2020/4085617

**Published:** 2020-02-11

**Authors:** Masahito Kawabori, Aki Tanimori, Shinri Kitta, Hideo Shichinohe, Kiyohiro Houkin

**Affiliations:** Department of Neurosurgery and Neurological Cell Therapy, Hokkaido University Graduate School of Medicine, Sapporo, Hokkaido, Japan

## Abstract

Cell therapy for central nervous system (CNS) disorders is beginning to prove its safety and efficiency. Intraparenchymal transplantation can be an option for cell delivery; however, one concern regarding this method is that the transplantation cannula may cause additional brain injuries. These include vessel damage, which results in brain hemorrhage, and clogging of the cannula by brain debris and/or cell clusters, which requires replacement of the cannula or forced injection causing jet flow of the cell suspension. We compared cannulas for cell delivery used in clinical trials, the Pittsburg and Mizuho cannulas, to a newly designed one, MK01, to assess their usability. MK01 has a spherical-shaped tip with a fan-like open orifice on the side of the cannula, which prevents vessel damage, clogging of brain debris, and jet flow phenomenon. We compared the extent of rat cervical and abdominal arterial damage with the cannula, the amount of debris in the cannula, the force needed to cause jet flow, and cell viability. While the viability of cells passed through the cannulas was almost the same among cannulas (approximately 95%), the Pittsburg cannula caused cervical arterial injury and subsequent hemorrhage, as it required a significantly smaller force to penetrate the arterial wall. Moreover, the Pittsburg cannula, but not the Mizuho and MK01 cannulas, showed high frequency of brain debris in the needle tip (approximately 80%) after brain puncture. While jet flow of the injection liquid was observed even when using smaller forces in the Pittsburg and Mizuho cannulas, MK01 constantly showed low jet flow occurrence. Thus, MK01 seems to be safer than the previously reported cannulas, although further investigation is necessary to validate its safety for clinical use.

## 1. Introduction

Transplantation of stem cells for central nervous system (CNS) disease has shown promising results in the laboratory [1–3], and several clinical trials are beginning to prove its safety and efficacy [4–7]. Although, an ideal transplantation route has not been determined yet, intraparenchymal transplantation remains an option for cell delivery, since it enables delivery of a sufficient amount of cells into the damaged brain compared to that achieved with intravascular transplantation [6, 8, 9]. To date, no cannula has been approved for cell transplantation by regulatory agencies, including the FDA, and different types of cannulas specially designed or adopted from other uses are being used for clinical trials [6, 10]. However, one main concern of direct transplantation is the potential risk of brain injury by the transplantation cannula. The risk of brain hemorrhage by electrode insertion during deep brain stimulation surgery is reported to be at approximately 2-5%, throughout the procedure [11], and stem cell transplantation presumably has similar or higher risk of such complication, as the cannula is inserted into brain areas with preexisting damages [6, 12]. Another concern is that brain debris and cell suspension may cause clogging of the cannula, since most stem cell types have adherence properties, and the cells are kept in a stabilized position for a while after delivery to the operation room. When cells are clogged in the cannula, the surgeon needs to change the cannula, thus causing additional damage to the brain, or push the syringe stronger to break the cell cluster, causing cell death and/or jet flow of the cell suspension, which can also cause additional brain damage. In order to overcome these problems, we developed a novel transplantation cannula, with a spherical-shaped tip and a fan-like-shaped open orifice on the side. In this report, we evaluated the design and performance of this novel cannula in comparison with cannulas used in clinical trials.

## 2. Material and Methods

### 2.1. Experimental Ethics

All animal experimental protocols were approved by the Animal Studies Ethical Committee at Hokkaido University Graduate School of Medicine (reference number 17-0066), and all animal procedures used in the present study were performed in accordance with the institutional guidelines for animal experimentation, the Guidelines for Proper Conduct of Animal Experiments by the Science Council of Japan, and the ARRIVE (Animal Research: Reporting In Vivo Experiments) guidelines. All human experiments including the stem cell and platelet harvesting were conducted in accordance with the guideline principles of the Declaration of Helsinki, and the study was approved by the institutional review board (reference number 012-0334). All participants provided written informed consent to participate in this study.

### 2.2. Cell Delivery Cannulas

**Figure 1:**
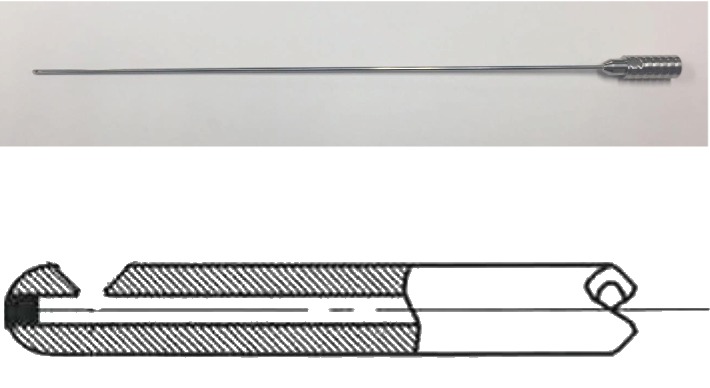
Photograph of the MK01 cannula and of the shape of the open orifice. The distal side of the opening orifice is curved just proximal to the equator line of the spherical tip so that the opening orifice does not appear from the inserting trajectory view.

Pittsburgh cell implantation cannula and stylet (SB2023, Synergetics, O'Fallon, USA), Mizuho Biopsy/Injection needle (MES-CG07-200-01, Mizuho, Tokyo, Japan), and our newly designed cannula, MK01, were examined in this experiment. The Pittsburg and Mizuho cannulas have been used in clinical trials for injecting stem cells into the brain [6, 10, 13]. The basic parameters of the cannulas are listed in Table 1. Briefly, Pittsburgh cannula's internal bore diameter is 250 *μ*m, and the cell ejection orifice is located at the end of the cannula, where the tip shows a slight narrowing of the outer diameter. Mizuho needle's internal bore is 300 *μ*m, and the cell ejection orifice is located on the side of the needle, 7 mm from the distal end. MK01 was modified from the Mizuho cannula and has the following characteristics (Figure 1): it is made of 100% stainless steel, the outer diameter of the cannula is 1.5 mm, and the internal bore diameter is 300 *μ*m, which enables direct attachment to the Leksell stereotactic arc system (Model G, Elekta, Sweden). The internal volume of the cannula is 23.9 *μ*L, and the instrument length is 19 cm, without flexibility. A characteristic feature of MK01 is that its ejection hole is located at the side of the cannula rather than the tip of the cannula. Furthermore, the ejection hole is designed to spread out in a fan-like form to avoid jet flow, even at high pressures. Moreover, the tip of the cannula is spherically shaped to avoid vessel injury during puncture and insertion. The distal side of the opening orifice is curved just proximal to the equator line of the spherical tip so that the opening orifice does not appear from the inserting trajectory view. The cannula can be sterilized using a conventional steam technique.

### 2.3. Experimental Animals

Eight-week-old male Sprague-Dawley rats (CLEA Japan, Inc., Tokyo, Japan), weighing 260–300 g, were used for experiments. Animals were housed in a controlled environment (25°C temperature, 50% humidity, and 12-hour light–dark cycle) and allowed free access to food and water. Rats were anaesthetized with isoflurane at an initial concentration of 4.0% in 70% N_2_O and 30% O_2_ gas, and anesthesia was maintained at a concentration of 2.0%, through a facial mask, as previously reported [2]. The rectal temperature of experimental animals was maintained between 36.5 and 37.5°C throughout the procedures using an automated heat pad. Animals were euthanized by deep anesthesia as previously mentioned using 5.0% isoflurane followed by cervical dislocation in a humane manner.

### 2.4. Cell Culture

Human bone marrow stem cell (BMSC) and platelet lysate (PL) were harvested from three healthy donors, as previously reported [10]. Briefly, bone marrow mononuclear cells were isolated via density-gradient centrifugation in Ficoll-Hypaque® (Pharmacia, Uppsala, Sweden), and cells were seeded in a 175 cm^2^ uncoated flask (EasYFlask 159910; Nunc) at a density of approximately 20 × 10^5^ cells/cm^2^ in 25 mL *α*-minimum essential medium containing 10% PL derived from healthy volunteers and 40 *μ*g/mL of gentamicin sulfate. After 24 h, nonadherent cells were removed by changing the medium. The culture medium was replaced twice a week. BMSCs were passed two or three times prior to using them for the subsequent procedure. BMSCs in the flasks were collected using TrypLe Select® (a recombinant trypsin substitute, Gibco) and centrifuged. The supernatant was decanted, and the cells were gently resuspended in ARTCEREB® (irrigation and perfusion solution used for cerebrospinal surgery; Otsuka Pharmaceutical Factory, Inc., Naruto, Japan) to a concentration of 5 × 10^7^ cells/mL. The size of the cells was measured using an automated cell size calculator (iSpect DIA-10, Shimadzu Co., Kyoto, Japan).

### 2.5. Cell Viability Analysis

Cell viability before and after passing the cannula was examined using a LUNA automated cell counter (Logos Biosystems, Korea), according to the manufacturer's instructions. Four different procedures were performed, and the relative ratio of cell viability before and after cell injection was calculated.

### 2.6. Vascular Puncture Experiment

After induction of anesthesia, the bilateral common carotid arteries (CCA) were exposed through a ventral midline incision of the neck. A rubber sheet was placed under the CCA to stabilize them. The cannula tip was placed on top of the artery by applying gentle pressure to see whether the cannula could penetrate the artery. If the artery was damaged, gentle compression of the artery for about 10 minutes enabled stopping of the arterial bleeding. Ten procedures were performed for each cannula at a different arterial position. After the procedure, rats were euthanized, and the abdominal aorta was harvested (approximately 7-10 mm length can be harvested). The arterial tube was opened and placed on the plastic sheet to cover a 3 mm hole in the plastic. The artery was stabilized on the plastic sheet by pins, which were hexagonally nailed at 1 mm from the plastic edge. Cannulas were attached using a digital force gauge monitor (ZTA-50N, IMADA, Toyohashi, Japan), and the power to penetrate the arterial wall was measured. Six different procedures were performed for each cannula (*n* = 3 for each experiment and two punctures per animal).

### 2.7. Brain and Liver Puncture Experiment

After induction of anesthesia, animals were fixed to a stereotactic apparatus. The skull was exposed, and a 2 mm diameter burr hole was made 3 mm right and left to the bregma, using a small dental drill, as previously reported [1]. The cannulas were slowly inserted into the brain parenchyma until the tip reached the skull base (approximately 10 mm from the surface of the dura matter). After withdrawing the cannula from the brain, the cannula was flushed with saline to assess the presence of brain debris present in the cannula. Three independent experiments consisting of 10 punctures were performed for each cannula (*n* = 3 for each experiment and ten punctures per animal). After the brain experiment, the abdomen was exposed by a linear skin incision. Then, the cannula was slowly inserted into the liver, 5 mm from the surface, and the cannula was flushed again to check the presence of debris as mentioned above.

### 2.8. Jet Flow Experiment

The cannulas were attached using an automated injection pump, and saline with 2% trypan blue was continuously injected at various injection speeds ranging from 0 to 11 mL/min. The cannula opening orifice was placed horizontally 10 cm above the floor, and the distance reached by saline was compared among 10 experiments for each cannula. Then, the cannula holes were vertically placed and attached using an 1 mL syringe containing saline with 2% trypan blue. The syringe, attached by a digital force gauge, was pushed to assess the force needed to cause a liquid fountain. Then, a phantom brain gel was prepared by 33% borax, as previously described with modifications [14]. The cannula was placed in the gel, 3 cm below the surface, and 2% trypan blue was injected at various speeds for 30 seconds. The shape of the injected saline was photographed to check the presence of jet flow.

### 2.9. Statistics

All data are expressed either as mean ± standard deviation or as median (interquartile range (IQR)). All statistical analyses were performed using JMP version 12 (SAS Institute Inc., Cary, NC, USA). Continuous data were compared by one-factor analysis of variance followed by the Tukey HSD test. Sample sizes were selected based on preliminary experiments. Briefly, in a one-way ANOVA study, sample sizes of 2 are obtained from the 3 groups whose means are to be compared. The total sample of 6 or 10 subjects achieves 100% power to detect differences among the means versus the alternative of equal means using an *F* test with a 0.0500 significance level. The size of the variation in the means is represented by their standard deviation which is 0.83. The common standard deviation within a group is assumed to be 1.00. We used PASS 14.0.9 (PASS Software by NCSS, LLC) to compute the statistical power. *p* values < 0.05 were considered statistically significant.

## 3. Results

### 3.1. No Difference in Cell Viability among Different Cannulas

Freshly prepared human BMSCs were passed through the cannulas. Median cell size was 30 mm, (IQR 20-70 mm). Cell viability with the Pittsburg, Mizuho, and MK01 cannulas was 95.8 ± 8.1%, 93.8 ± 8.8%, and 94.1 ± 9.9%, respectively. However, occlusion of the cannula by cell clusters occurred once in the four attempts using the Mizuho cannula.

### 3.2. High Frequency of Arterial Vessel Damage with the Pittsburg Cannula

**Figure 2:**
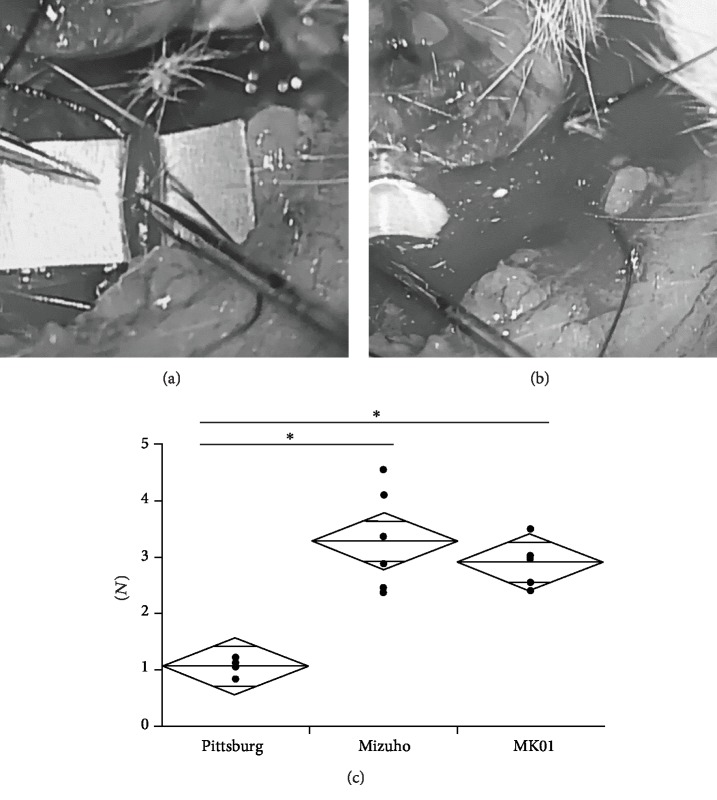
Photographs of compressing the cervical artery using the Pittsburg cannula (a) and of massive hemorrhage after arterial vessel penetration (b). Graph showing the force needed to penetrate the abdominal arterial wall with each cannula (c). The Pittsburg cannula required a significantly smaller force to penetrate the artery compared with the Mizuho and MK01 cannulas.

When gently pressing the artery with each cannula, only the Pittsburg cannula penetrated the artery and resulted in massive hemorrhage (Figures 2(a) and 2(b)). However, the Mizuho and MK01cannulas did not cause arterial vessel rupture. The force needed to penetrate the abdominal arterial wall was significantly lower with the Pittsburg cannula (1.06 ± 0.13 N) than with the Mizuho (3.28 ± 0.89 N) and MK01 (2.91 ± 0.38 N) cannulas (Figure 2(c)).

### 3.3. Brain Debris Is Found in the Pittsburg Cannula

**Figure 3:**
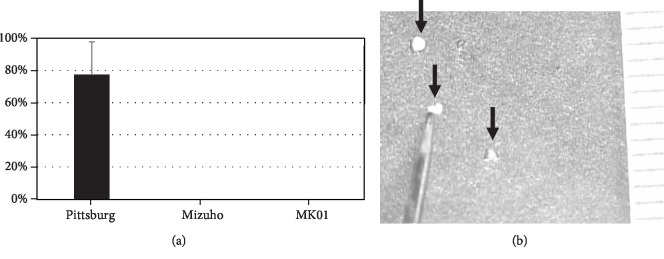
Amount of brain debris found in each cannula (a) and representative figure of the debris (b, arrow).

Brain debris was frequently found only in the Pittsburg cannula (77.1 ± 20.6%), while there was no debris in the Mizuho and MK01 cannulas (Figures 3(a) and 3(b)). There was no liver debris found in any of the needles.

### 3.4. MK01 Has the Lowest Capacity of Jet Flow

**Figure 4:**
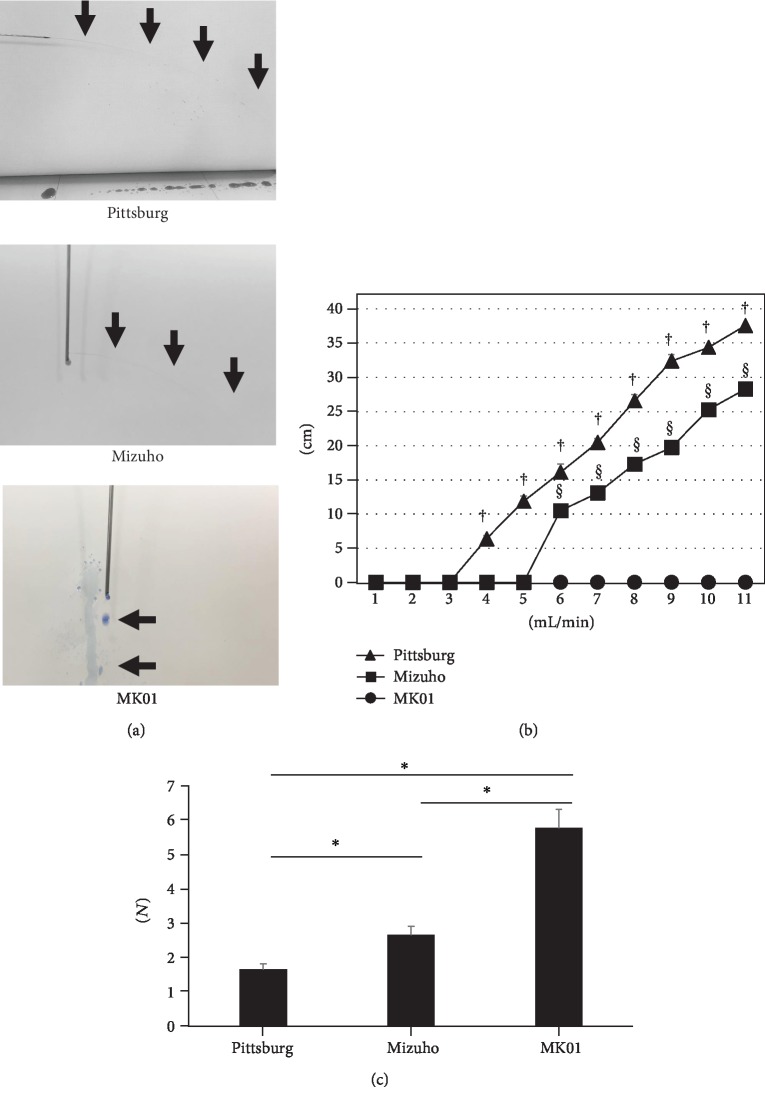
Representative figures of the jet flow with each cannula (a, arrow) and the distance reached by saline (b) (^†,§^*p* < 0.05 vs. MK01). The force (N) needed to cause vertical jet flow (fountain) (c) (^∗^*p* < 0.05).

**Figure 5:**
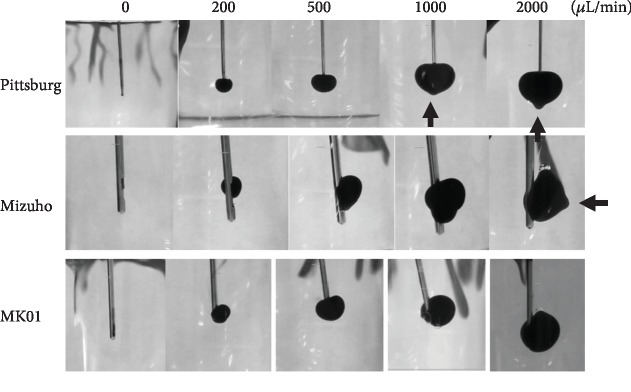
Colored saline injected in the gel imitating the brain. The Pittsburg and Mizuho needles showed jet flow prominence (arrow) at 2000 *μ*L/min (arrow), while MK01 did not cause protuberance.

When the needle holes were horizontally placed and injected with colored saline, all showed dripping of the liquid at a rate between 0 and 3 mL/min; however, the Pittsburg cannula started to show horizontal jet flow at 4 mL/min, with the flow distance continuing to expand as the injection speed increased. The Mizuho needle also started to show jet flow from 5 mL/min, with the distance also expanding at higher speeds. However, MK01 did not show jet flow for speeds up to 11 mL/min (Figures 4(a) and 4(b)). We then checked the force needed to cause jet fountain by pushing the needle using a digital force gauge monitor. There were significant differences among the three cannulas, with the Pittsburg cannula requiring a significantly smaller force (1.62 + 0.18 N), followed by the Mizuho cannula (2.62 ± 0.27 N), while MK01 showed the highest resistance force needed against the jet flow (5.76 ± 0.55 N) (Figure 4(c)). When colored saline was injected into the gel imitating the brain, the Pittsburg and Mizuho cannulas showed protrusion of the injected saline at speeds of 1000 and 2000 *μ*L/min, while MK01 constantly showed spherical injection (Figure 5).

## 4. Discussion

This study demonstrates that the newly developed MK01 cannula significantly reduces the occurrence of vessel injury and jet flow phenomenon, while preserving injected cell viability, compared with the Pittsburg and Mizuho cannulas.

Stem cell-based therapy is emerging as a promising treatment for a variety of neurological diseases and injuries. There are several routes currently available for cell transplantation, including intraparenchymal, intravenous, intra-arterial, and intrathecal injections. Although the optimal cell transplantation route has not been determined, most clinical studies have applied intraparenchymal or intravenous routes of transplantation [15]. The intraparenchymal route delivers a sufficient number of cells into the desired brain lesion, having a strong advantage over the intravenous route, by which only a small number of cells can be delivered [2], most of which are trapped in the lung and spleen. The intravenous delivery method is still efficient because neurotrophic factors are released from the cells and are critical in immunomodulation and anti-inflammation, especially at the acute stage of disease. However, when considering the subacute and chronic stages, delivering cells to the damaged area needs to be important to reorganize the damaged neurons, and recent systemic analyses have reported that intraparenchymal cell transplantation is superior to the intravenous one from an efficacy point of view, as demonstrated in both the preclinical and clinical trials [15]. Nevertheless, the intraparenchymal transplantation has a serious burden, which is that the transplantation cannula can induce intraparenchymal hemorrhage, and occlusion of the cannula may occur.

Intraparenchymal hemorrhage by the cannula is a serious complication. Deep brain stimulation procedures are shown to have approximately 5% of brain hemorrhage caused by the stimulation cannulas, while ventricle drainage for hydrocephalus results in 1.5% mortality related to lobar hemorrhages along the catheter track. One clinical trial reported one subdural hematoma in 18 patients who underwent intraparenchymal stem cell transplantation using the Pittsburg cannula [6] and one in 13 patients who underwent intraparenchymal human neural stem cell transplantation [8]. In contrast, Levi et al. showed no needle (30-gauge) complication when injecting human CNS stem cells in 29 patients with cervical and thoracic cord injury, with four to eight sites and a depth of 3-4 mm from the spinal cord surface being selected for injection [16]. However, the possibility of intraparenchymal hemorrhage caused by the injection cannula should not be underestimated, as it may cause catastrophic complications. The Pittsburg cannula, which we used in this study, has an opening orifice with a narrow-shaped tip, which may injure the vessel during the insertion procedure. In contrast, the Mizuho needle has a flat tip which is safer but still may cause vessel damage when attached to the vessel. Thus, we used a newly developed cannula with a round-shaped tip and an opening orifice beyond the edge of the spherical equator, ensuring that the spherical shape is preserved from the trajectory (barrel) view.

Cannula obstruction is another complication that should always be considered [17, 18]. Brain debris or cell clusters can cause obstruction of the cannula. Indeed, we found cannula obstruction in one out of four trials during cell survival examination, as well as a very high percentage of brain debris when using the Pittsburg cannula. In addition, we have encountered cannula obstruction in 3 out of 7 patients with stem cell transplantation using the Mizuho cannula (unpublished data) [10]. When the cannula is obstructed, the surgeon may push the syringe a little stronger or may need to replace the cannula. When the syringe is pushed stronger in order to break cell clusters, this may cause a strong force to the cells or cell suspension, resulting in cell death and/or intraparenchymal jet flow of the cell suspension. Jet flow of the cell suspension may in turn cause additional damage to the brain, considering that jet flow is actually applied for brain tumor resection [19, 20]. The cannula that we developed here has a fan-like-shaped opening orifice. Fan-like-shaped orifices are shown to produce turbulence flow when the liquid emerges from them, while turbulence flow decreases the projection speed and prohibits the translator movement of the projecting substances [21].

There are some limitations in this study. First, the evaluation of the needle is mostly done in the in vitro study. When considering brain hemorrhage, the needles compared in this study are too large for the rodent brain to consider. Larger animals would be appropriate for evaluation of this needle for monitoring the occurrence of brain hemorrhage, but it would not be suitable from the animal welfare point of view. Second, the reason for cannula obstruction is not fully examined. The Mizuho cannula frequently showed the obstruction in this experiment as well as in the clinical trial, but not with MK01 which has almost the same internal shape. The curve at the end of the needle may be one reason for the difference, but further evaluation would be necessary to achieve better cannula development.

## 5. Conclusions

We developed a safer cannula for intraparenchymal cell transplantation, which has a spherical tip with a fan-like opening orifice on the side. The new cannula decreases the incidence of vessel damage and jet flow phenomenon and is safer than the previously reported cannulas, though further investigation is necessary to validate its safety for clinical use.

## Figures and Tables

**Table 1:** Basic characteristics of the cannulas.

		Outer diameter	Internal bore diameter	Internal volume	Flexibility	Injection hole	Shape of the tip
Pittsburg	Stainless steel	890 mm	250 mm	20 mL	Yes	Tip	Narrowed with open tip
Mizuho	Stainless steel	1500 mm	300 mm	23.9 *μ*L	No	Side	Flattened
MK01	Stainless steel	1500 mm	300 mm	23.9 *μ*L	No	Side	Spherical

## Data Availability

Raw data were generated at Hokkaido University. Derived data supporting the findings of this study are available from the corresponding author (MK) on reasonable request.
