# Corrigendum to “Evaluation of Novel Stereotactic Cannula for Stem Cell Transplantation against Central Nervous System Disease”

**DOI:** 10.1155/2021/9796010

**Published:** 2021-04-29

**Authors:** Masahito Kawabori, Aki Tanimori, Shinri Kitta, Hideo Shichinohe, Kiyohiro Houkin

**Affiliations:** Department of Neurosurgery and Neurological Cell Therapy, Hokkaido University Graduate School of Medicine, Sapporo, Hokkaido, Japan

In the article titled “Evaluation of Novel Stereotactic Cannula for Stem Cell Transplantation against Central Nervous System Disease” [1], there was an error in Table 1, where the unit for the outer diameter and internal bore diameter columns should be corrected to “*μ*m.” In the internal volume column, the value “20 mL” should be corrected to “20 *μ*L.”

The corrected version of Table 1 is as follows:

The authors confirm that this does not affect the results and conclusions of the article, and the editorial board agrees to the publication of a corrigendum.

## Figures and Tables

**Table 1:** Basic characteristics of the cannulas.

		Outer diameter	Internal bore diameter	Internal volume	Flexibility	Injection hole	Shape of the tip
Pittsburg	Stainless steel	890 *μ*m	250 *μ*m	20 *μ*L	Yes	Tip	Narrowed with open tip
Mizuho	Stainless steel	1500 *μ*m	300 *μ*m	23.9 *μ*L	No	Side	Flattened
MK01	Stainless steel	1500 *μ*m	300 *μ*m	23.9 *μ*L	No	Side	Spherical
